# Surgical decision making in late-presenting Hirschsprung's disease: direct pull-though or stoma first

**DOI:** 10.3389/fsurg.2026.1773691

**Published:** 2026-03-04

**Authors:** Ahmed Arafa, Ahmed S. Ragab, Abdelhalem Showkat Mohamed, Mahmoud Tarek Mohamed, Ahmed E. Arafat, Abdelhafeez Mohamed Abdelhafeez

**Affiliations:** 1 Pediatric Surgery Department, Faculty of Medicine, Cairo University, Cairo, Egypt; 2 Pediatric Surgery Department, Faculty of Medicine, Port Said University, Port Said, Egypt; 3 Pediatric Surgery Department, Faculty of Medicine, Menia University, Menia, Egypt; 4 Pediatric Surgery Department, Faculty of Medicine, Beni Suef University, Beni Suef, Egypt

**Keywords:** Duhamel, Hirshsprung's disease, staging, stoma, Swensons

## Abstract

**Aim of the study:**

To evaluate the use of staged vs. one-stage surgical management for Hirschsprung's disease (HSD) in older children.

**Methods:**

In total, 30 patients were diagnosed with HSD and all cases were confirmed by rectal biopsy. For treatment, rectal irrigation was carried out for two months to achieve colonic decompression; treatment failure occurred in 15 cases due to decompressed colons. In these cases, colostomy or ileostomy were carried out according to the distal or proximal site of the transitional zone to the transverse colon. After two months, eight cases of Laparoscopic Duhamel and seven cases of laparoscopic-aided Swenson were performed.

**Results:**

An anal dilation program was done three weeks after pull-through. Postoperatively, there were three cases of Swenson cases stenosis and two cases of enterocolitis that responded to conservative treatment and one case of fecal incontinence. In Duhamel cases, we had three cases of constipation and three cases of enterocolitis, with no anastomotic leak cases. Four cases of stomal prolapse and skin excoriation occurred.

**Conclusion:**

Both staged and single-staged procedures are safe options for the management of hirshspung's disease in older children.

## Introduction

Contrary to previous reports suggesting that laparoscopic pull-through is universally feasible, our cohort demonstrates that a primary stoma remains necessary in late-presenting cases involving severe colonic dilation or clinical instability. In our previous published work, our surgical strategy was based on the principle that laparoscopic pull-through was feasible for all cases, considering that most children reached surgery after adequate preoperative preparation, which allowed acceptable decompression of the dilated colon.

In this current study, however, some children showed marked colonic dilatation or insufficient response to preoperative bowel preparation, making a direct one-stage laparoscopic pull-through potentially unsafe. In such selected cases, the more appropriate initial intervention was to perform a primary stoma (colostomy or ileostomy) first, followed later by the definitive pull-through procedure.

Thus, the fundamental difference between the two papers is:

Previous paper:

- Concluded that all patients can undergo laparoscopic pull-through without diversion
- Surgical decision was predominantly one-stage
- Aim was to confirm laparoscopic effectiveness in all cases

Current paper

- Demonstrates that some late-presenting patients require a primary stoma first to ensure a safe and successful pull-through
- Surgical decision was individualized depending on the degree of dilatation and the response to preoperative preparation and sometimes involved two stages
- Aim was to compare direct laparoscopic pull-through vs. initial stoma followed by pull-through in late-presenting patients

This modification reflects real-world, patient-specific surgical decision-making and advances treatment planning without duplicating our earlier findings. Furthermore, it addresses a critical clinical gap not covered in our previous publication. Hirschsprung disease is most often identified in infancy, although presentation may be delayed until later childhood in some cases (1). It is characterized by the absence of enteric ganglion cells over a segment of the bowel, most commonly involving the rectosigmoid colon. Surgical management aims to resect the aganglionic segment and restore continuity; many centers now employ minimally invasive, single-stage pull-through techniques (e.g., Duhamel, Soave, Swenson), yet staged approaches with a proximal diversion remain necessary in selected patients with marked colonic dilatation or clinical instability (2–5).

## Methods

The study included 30 patients diagnosed with HSD (17 males and 13 females), all of whom were over three years of age. Hirschsprung-associated enterocolitis (HAEC) was diagnosed based on clinical and radiological findings (6–8). Patients with mild to moderate (minor) signs of enterocolitis who responded to conservative management were included. Patients with severe (major) enterocolitis who failed to respond to conservative treatment and required urgent surgical intervention were excluded from the study.

Minor signs included mild abdominal distension, diarrhea, low-grade fever, and mild radiological bowel dilatation. Severe HAEC was defined by systemic toxicity, severe abdominal distension, or failure of conservative management.

So, we excluded cases of intestinal obstruction or enterocolitis that did not respond to conservative treatment and were in urgent need of stoma.

All surgical procedures, including direct pull-through or stoma formation followed by definitive surgery, were performed by experienced pediatric surgeons at our institution following a standardized surgical protocol.

All procedures were performed by consultant pediatric surgeons with experience in managing Hirschsprung disease.

All patients received a preoperative regimen consisting of rectal irrigation and oral fluid intake over approximately two months to reduce colonic dilatation. Those who did not achieve adequate decompression proceeded to proximal diversion (ileostomy or colostomy) according to the level of transition. The preoperative regimen and diversion strategy followed established protocols for late-presenting Hirschsprung disease (9).

### A. Colonic diameter ratio (CDR)

Using contrast enema, we measured the maximal dilated colonic diameter (D) and the normal colonic diameter (N), the latter of which is typically measured in the descending colon above the transition zone.

The Colonic Diameter Ratio (CDR) was calculated as

CDR=D/N

Interpretation:

CDR ≤1.5–2.0: Suitable for primary pull-through.
CDR >2.5: High risk; stoma recommended.
CDR >3.0: Strong contraindication to primary pull-through.

Objective colonic regression was assessed using the contrast enema-derived CDR and the absolute colonic diameter. Persistent dilation (CDR >2.5) despite 8 weeks of adequate rectal irrigation was considered failure of decompression, and staged surgery was performed instead.

This study is reproducible and non-subjective.

Because normal colonic diameter varies substantially with age, colonic dilation was assessed using relative diameter differences rather than absolute colonic size. Comparison of dilated and normal bowel segments within the same patient allowed for an age-independent and objective evaluation of colonic regression.

In late-presenting Hirschsprung's disease, surgical decision-making cannot rely on a single objective parameter. Although the dilated-to-“normal” colon ratio has been traditionally used as a radiologic guide, its reliability is limited, as the colon in older children is rarely truly normal and often exhibits hypertrophy, fibrosis, or reduced compliance. Therefore, in the present study, the ratio was used only as an adjunctive reference rather than a definitive decision-making criterion. Final surgical strategy was determined based on a combination of functional response to decompression, intraoperative bowel wall characteristics, anatomical extent of aganglionosis, and the child's overall clinical condition.

If severe intestinal distension had not yet occurred and the patient was not responding to the regimen, ileostomy or colostomy was done according to the transitional zone site.

If the transitional zone was proximal to the transverse colon, ileostomy was carried out.

If it was distal to the transverse colon, colostomy was carried out.

If the dilated intestine regressed, we performed a colonic pull-through after excision of the aganglionic colon at stage one without stoma; this was in the form of a laparoscopic-aided Swenson in seven cases and a laparoscopic-aided Duhamel in eight cases.

### Laparoscopic ileostomy or colostomy

**Figure 1:**
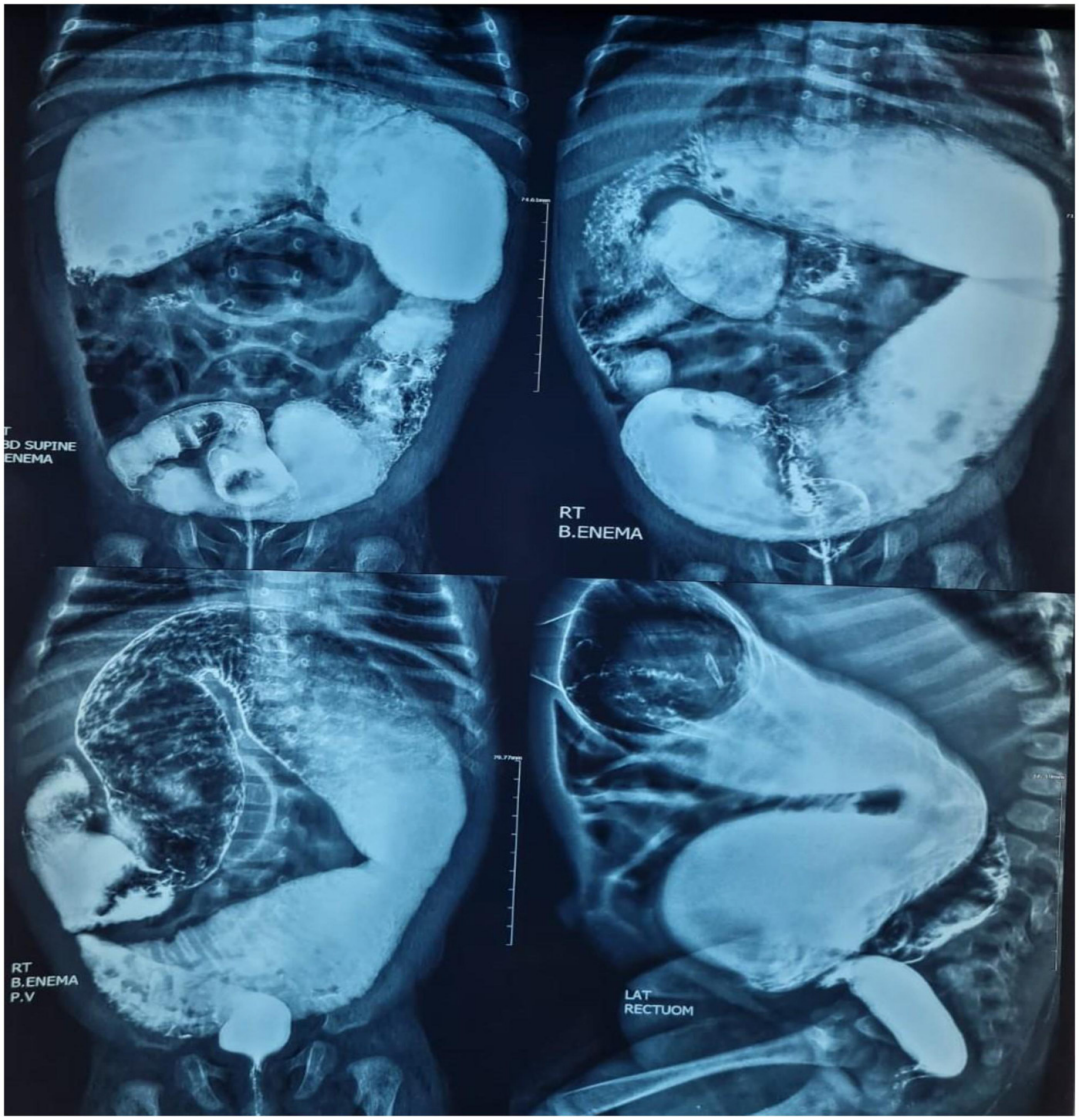
Barium enema: severely dilated colon before irrigation regimen.

**Figure 2:**
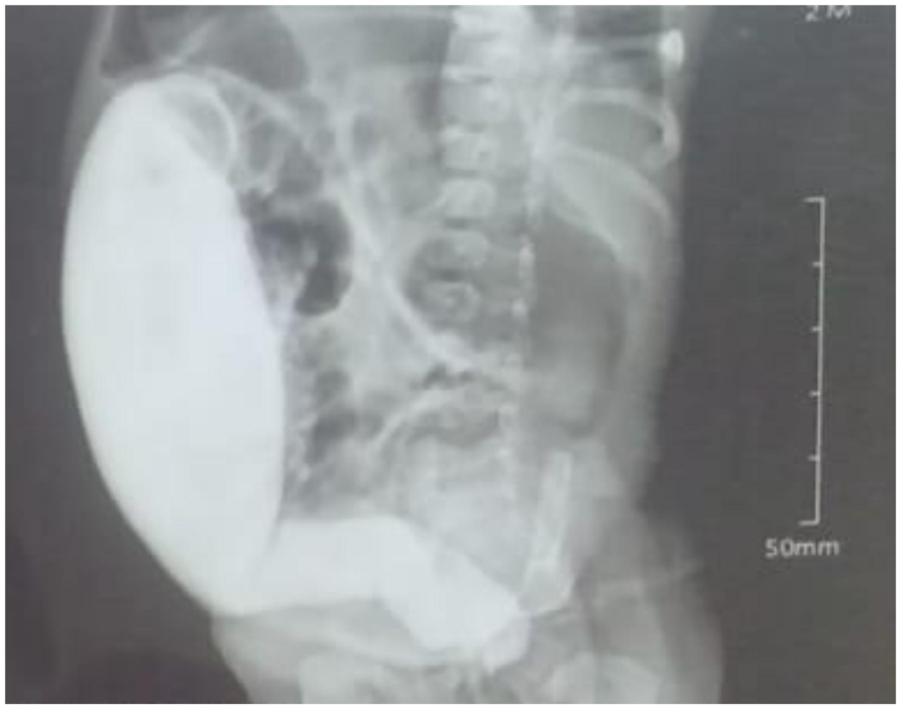
Barium enema: regressed dilated colon after the rectal dilation regimen.

Figures 1, 2 display a colon dilated by barium enema after rectal irrigation regimen for 2 months (Figures 1, 2).

Laparoscopic colostomy or ileostomy was done after making sure the severely dilated colon had not regressed.

Laparoscopic evaluation of the dilated colon was performed to decide whether stoma or the one-stage procedure should be done using multiple ascending fresh-frozen biopsies, especially in cases with ill-defined funnels, to determine aganglionic level.

Laparoscopic ileostomy or colostomy was done according to the transitional zone site: if it was proximal to the transverse colon a simple loop ileostomy was done; if it was distal to the transverse colon, a sigmoid colostomy was performed above the funnel.

Two months after the initial colostomy or ileostomy, the definitive procedure was performed.

**Figure 3:**
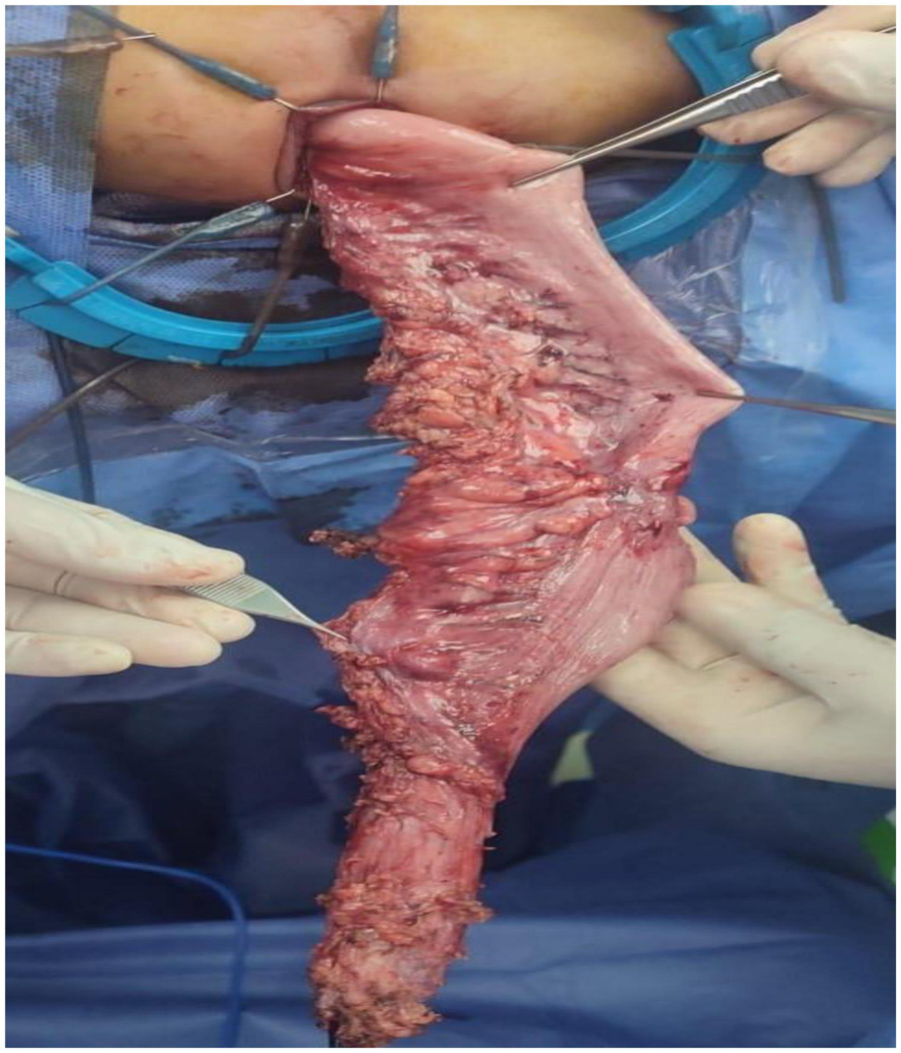
Transanal aided Swenson after laparoscopic dissection of the distal colon.

Laparoscopic-assisted Swenson procedure (Figure 3):

- Laparoscopic devascularization of the distal colon and mobilization of the ganglionated colon.
- Transanal dissection performed in close proximity to the rectal wall.
- Coloanal anastomosis created 1 cm above the dentate line.

**Figure 4:**
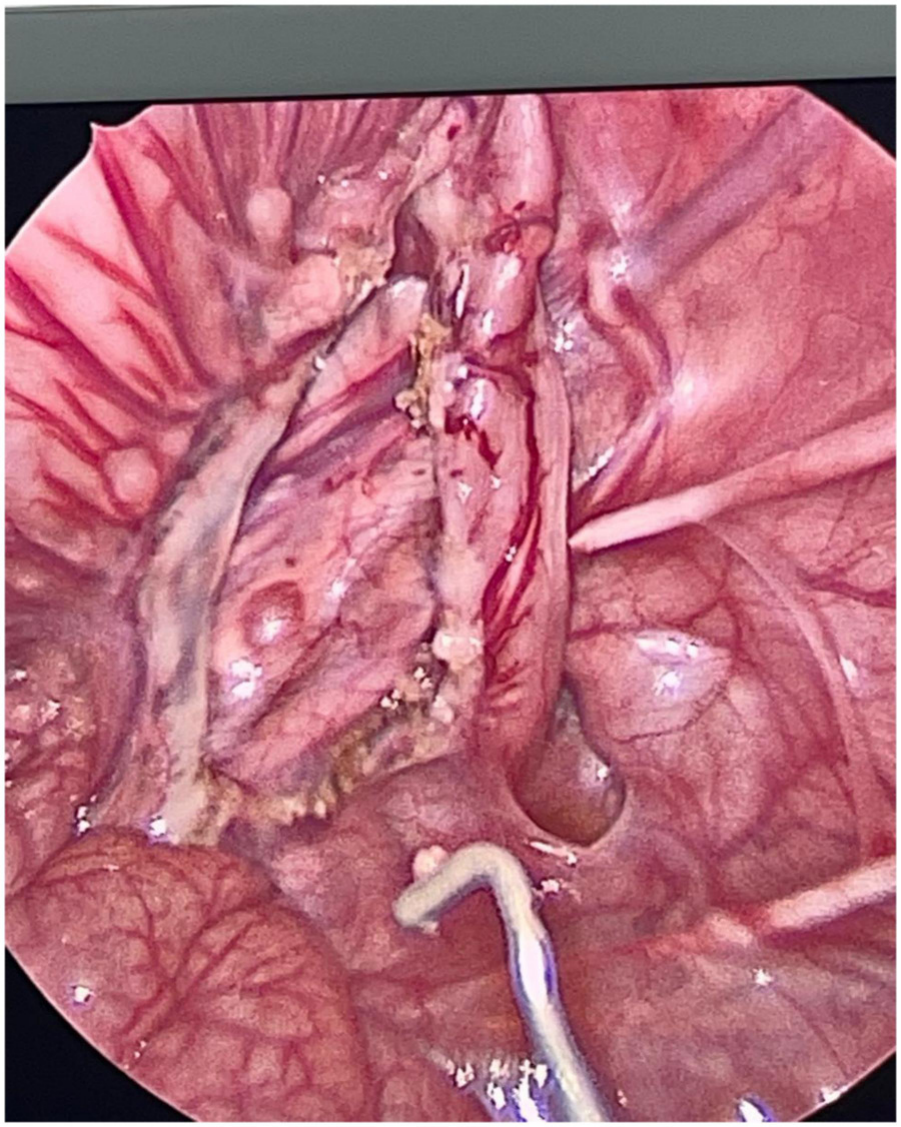
Dissection of only the posterior part of the rectum for the Duhamel procedure.

Laparoscopic-aided Duhamel:

- Laparoscopic devascularization of the distal colon, with dissection of only the posterior part of the distal colon (Figure 4).
- Mobilization of the ganglionated colon
- Transanal dissection of the posterior wall of the rectum, with transanal neocolon pull-through done posteriorly to the rectum
- Anterior Colorectal anastomosis performed using GIA stabler 55, then anastomosis completed 1 cm above the dentate line.

## Results

An anal dilation program was initiated three weeks after the pull-through procedure. Among the Swenson cases, three patients developed stenosis that responded to regular dilation. Additionally, two cases of postoperative enterocolitis were successfully managed with conservative treatment, namely parenteral antibiotics, rectal irrigation, intravenous fluids, and NPO status, and one case of fecal incontinence was reported. For Duhamel cases, we had three cases of constipation and three cases of enterocolitis; there were no cases of anastomotic leak.

There were four cases of stomal prolapse, and skin excoriation occurred in all operations.

## Discussion

Chan et al. (2) reported 41 cases with a median age of 9.9 years, whereas Nam et al. (10) reported a median age of 3 years among 8 children, which is consistent with our study. Male predominance was reported by several authors [Maerzheuser S et al. (1), Baeza et al. (3)]; this was also reflected in our study, which had 17 male and 13 female patients.

Nasir et al. (11), reported associated neurologic, cardiovascular, urologic, and gastrointestinal abnormalities as well as a connecting with Down's syndrome, however, we excluded patients with Down's syndrome and cardiac anomalies from our study.

Maerzheuser S et al. (1), had 10 patients with late-diagnosed Hirschsprung disease; their main presentation was recurrent gastrointestinal infection but, in our cases, the main presentation was chronic constipation.

Full-thickness rectal biopsy remains the diagnostic gold standard and was performed in all patients in our series. Barium enema and laparoscopic inspection were used adjunctively to map the transition zone and guide the decision between single-stage pull-through and staged management—a decision influenced primarily by the degree of colonic dilatation after preoperative decompression (1, 2), Barium enema and laparoscope were also used to determine aganglionosis level and to detect if the operation could be completed in one stage or needed multiple stages, which also depended on the size of the dilated pull-through colon after the preoperative preparation regimen.

Chan et al. (2) and Nam et al. (10) used a one-stage laparoscopic-assisted endorectal pull-through for all patients following preoperative preparation with rectal irrigation under general anesthesia. Similarly, Baeza et al. (3) performed the Duhamel procedure in all eight of their cases. In contrast, the current study of 30 cases utilized both Duhamel and Swenson procedures, performed in either one or multiple stages.

Baeza et al. (3) and Mabula et al. (4) performed a colostomy on all their patients; they allowed time for the dilated colon to recover to reduce the incidence of postoperative enterocolitis.

In this study, the selective use of a primary stoma in our series reflects individualized, response-based surgical planning, representing an optimization of the laparoscopic pathway rather than a deviation from it.

This approach underscores clinical decision-making tailored to colonic decompression response and patient stability, enhancing pull-through safety without contradicting prior evidence supporting laparoscopic feasibility.

Stomas were performed only in cases refractory to rectal irrigation. Seven patients required a proximal ileostomy because the transition zone was proximal to the sigmoid colon. In the remaining eight cases, where the transition zone was distal to the sigmoid, a sigmoid colostomy was performed just above the funnel. Excision of the aganglionic segment and the pull-through procedure were then completed after two months. Eight cases of Laparoscopic Duhamel were done in one stage, while seven cases of Laparoscopic Swenson were done in one stage.

Mabula et al. (4), reported colostomy-related complications, skin excoriations, and colostomy prolapse in more than half of their patients, colostomy retraction in 16% of their patients, and parastomal hernia in 4%. In our study, we had four cases of stomal prolapse, and skin excoriation occurred in all operations, especially with ileostomy.

Tang et al. (5), had perianal excoriation in 32 cases, enterocolitis in 14 cases, anastomotic leakage in three cases, postoperative adhesive bowel obstruction in two cases (1.1%), anastomotic stenosis in four cases, and soiling in seven cases, while Chan et al. (2), had soiling in more than half of their cases, although no stricture was reported. Mabula et al. (4), reported fecal incontinence in only 2.1% of cases; in this study, we had three Swenson cases that responded to regular dilation, two cases of postoperative enterocolitis that responded to conservative treatment only in the form of parenteral antibiotics, rectal irrigation, intravenous fluids, and NP0, and one case of fecal incontinence. In our Duhamel cases, we had three cases of constipation and three cases of enterocolitis; there were no cases of anastomotic leak.

## Conclusion

Hirschsprung's disease typically presents early in life, making its occurrence in older children rare. Despite advancements in minimally invasive techniques that facilitate one-stage repairs, the management of late-presenting cases remains challenging and often necessitates a multi-stage approach.
